# Monolithic zirconia outperforms metal-ceramic in mechanical reliability for single implant crowns but lacks long-term validation

**DOI:** 10.1038/s41432-025-01137-4

**Published:** 2025-05-07

**Authors:** Kelvin I. Afrashtehfar

**Affiliations:** 1 Department of Reconstructive Dentistry and Gerodontology, School of Dental Medicine, University of Bern, Berne, Switzerland; 2 Oral Implantology Research Institute, Dubai, UAE; 3 Private Practice limited to Prosthodontics and Implant Dentistry, White Rock, Canada

**Keywords:** Dental implants, Fixed prosthodontics

## Abstract

**A Commentary on:**

**Malhotra T, Kumar Yadav B, Singh Phukela S** **et al***.*

A comparative evaluation of prosthetic and biologic outcomes as influenced by two different implant restorative materials: a prospective, split-mouth study. Int J Prosthodont. 2025; 10.11607/ijp.8729.

**Design:**

This was a prospective split-mouth study, where each patient received monolithic zirconia and metal-ceramic implant-supported crowns on contralateral sides in the same arch. This design guaranteed direct intra-patient comparison, reducing inter-individual variability.

**Case selection:**

Twenty partially edentulous patients (14 males, 6 females) were selected based on strict inclusion criteria, ensuring bilateral posterior implant placement with opposing natural dentition. Patients with parafunctional habits, active periodontal disease, or systemic conditions affecting bone metabolism were excluded.

**Study timeline:**

Implant placement and prosthetic restoration were performed per standard clinical protocols. The study assessed outcomes at baseline, 1-year, and 2-year follow-ups, measuring prosthetic integrity, periodontal health, and inflammatory markers.

**Data analysis:**

Clinical indices (plaque index, bleeding on probing, probing depth), peri-implant crevicular fluid biomarkers (MMP-8 levels), and prosthetic performance (USPHS criteria) were statistically analysed using chi-square tests, ANOVA, and Student t-tests, with significance set at *P* < 0.05.

**Results:**

Both materials showed 100% implant and prosthetic survival rate over 2 years. Metal-ceramic crowns exhibited higher incidences of ceramic chipping and screw loosening, while monolithic zirconia crowns demonstrated greater mechanical stability but poorer aesthetic match. No significant differences in marginal bone loss (MBL) or MMP-8 inflammatory marker levels were observed between groups. However, higher plaque index (PI) and probing depth (PD) were recorded for metal-ceramic crowns.

**Conclusions:**

Monolithic zirconia crowns demonstrate superior mechanical reliability and fewer technical complications but have aesthetic limitations compared to metal-ceramic crowns. Biologic outcomes were comparable between both materials. Clinicians should weigh mechanical durability versus aesthetic demands when selecting implant-supported restorations. Further long-term studies are recommended to validate these findings.

## GRADE Rating:



## Commentary

Implant-supported restorations have significantly advanced prosthodontic treatments, providing functional and aesthetic rehabilitation with high long-term survival rates. Metal-ceramic restorations have traditionally been considered the gold standard due to their mechanical durability and predictable aesthetics, with documented survival rates exceeding 95% over five years^1^. However, the high incidence of veneer chipping, framework fractures, and screw loosening in metal-ceramic crowns has fuelled the search for more durable alternatives. Monolithic zirconia, a high-strength ceramic with improved fracture resistance and lower bacterial adhesion, has emerged as a promising alternative^2^. Despite these advantages, concerns remain regarding its aesthetic limitations, wear properties against natural dentition, and potential phase transformation over time. Given these conflicting attributes, this study^3^ aimed to provide a direct comparative analysis of metal-ceramic and monolithic zirconia restorations in a controlled split-mouth setting, addressing both prosthetic and biologic factors influencing long-term success.

A major strength of this study laid in its split-mouth design, which eliminated inter-patient variability and allowed for a direct intra-patient comparison, increasing the internal validity of findings. The study also benefited from comprehensive outcome measures, including prosthetic integrity, peri-implant crevicular inflammatory markers, and periodontal indices, warranting a multi-faceted evaluation of each material. The statistical approach, incorporating ANOVA and chi-square tests, ensures appropriate handling of data variance and significance testing.

Despite these strengths, the study has several critical weaknesses that undermined its clinical applicability. The small sample size (*n* = 20) significantly limited statistical power and reduced generalisability to a broader patient population. The lack of blinding introduced potential observer bias, as both patients and evaluators were aware of material assignments, potentially affecting subjective assessments. Moreover, while the study evaluated mechanical stability, it failed to address long-term surface degradation and wear properties of zirconia, which have been a major concern in clinical practice^4^. Fundamentally, the two-year follow-up period, although longer than many preliminary reports, is insufficient to assess the long-term performance of implant restorations. Evidence-based dentistry considers outcomes below four years as short-term data, and such limited follow-up cannot fully capture mechanical degradation, aesthetic evolution, or biologic stability [6,12]. The absence of patient-reported outcomes (PROs), such as aesthetic satisfaction, perceived comfort, and functional adaptation, further limits its real-world applicability, as prosthetic success is not solely defined by mechanical performance^5^. Another overlooked aspect was the impact of opposing dentition, as zirconia has been shown to induce greater wear on enamel compared to metal-ceramic restorations.

To sum up, this study provided useful clinical data but lacked the methodological rigour and duration to be considered high-quality evidence. While the findings suggest that monolithic zirconia crowns outperform metal-ceramic restorations in terms of mechanical reliability and lower technical complications, their aesthetic inferiority and unknown long-term wear properties remain significant limitations. Metal-ceramic crowns continue to be the preferred choice for anterior regions, whereas zirconia could serve as an alternative for posterior restorations where mechanical stability is a priority. However, with a short-term follow-up of only two years, the study does not provide conclusive data on long-term degradation, material performance, or biologic stability. Given these methodological limitations, the evidence is insufficient to dictate clinical practice and instead should serve as a basis for further investigation. Larger randomised controlled trials (RCTs) with longer follow-up periods exceeding seven years, blinded assessments, and patient-reported outcome measures are needed to provide definitive guidance on material selection. Until such evidence emerges, clinicians must balance durability, aesthetics, and biologic compatibility when selecting implant-supported crowns, tailoring choices to individual patient needs and clinical circumstances.

Practice points

- Monolithic zirconia crowns offer superior mechanical reliability, making them preferable for posterior restorations, though their long-term wear properties remain uncertain.
- Metal-ceramic crowns provide better aesthetics but are more prone to technical failures such as ceramic chipping and screw loosening, making them more suitable for anterior restorations.
- Both materials show similar short-term biologic responses, but due to the study’s methodological limitations, stronger long-term evidence is needed before definitive clinical recommendations can be made.
